# Need for universal acceptance of preprinting by editors of journals of health professional education

**DOI:** 10.1007/s40037-022-00710-2

**Published:** 2022-04-25

**Authors:** Nena Schvaneveldt

**Affiliations:** Members of the Editorial Board of the Journal of the Academy of Health Science Educators: A Pre-print Repository (JAHSE:Pre) of the University of Utah, University of Utah Health Sciences, Salt Lake City, UT USA

To the Editor,

In their 2018 article, Maggio et al. [1] highlight the benefits of depositing manuscripts in preprint repositories, which include early discovery, access, and increased opportunities for feedback. Preprinting occurs when a manuscript is posted to a web-based, preprint repository prior to formal submission to a journal. However, our experience over the past 18 months reveals a lack of universal acceptance.

In 2020, we implemented a program to help faculty new to educational scholarship enhance their ability to disseminate their work. This program includes both peer coaches and a web-based preprint repository, known as the *Journal of the Academy of Health Science Educators: A Preprint Repository or JAHSE:PRE *(https://jahse.med.utah.edu/). Indeed, we have found that peer coaching while preparing a manuscript followed by the chance to preprint that manuscript to be particularly helpful for early career scholars or faculty new to education scholarship [2].

While publishers in multiple fields are adopting preprints [2], we have discovered a great deal of confusion about the pros and cons of preprinting as well as disparity in publishers’ policies regarding preprinting in health professions education (HPE). In seeking to resolve this confusion, we documented preprint policies at 74 journals within HPE (e.g. nursing, medicine, pharmacy, dentistry, rehabilitation sciences, nutrition). We culled preprint policies for 43 (58%) journals using journal websites, JISC’s Sherpa Romeo tool, and Wikipedia’s list of academic publishers by preprint policy. We then obtained information from email solicitations for an additional 27 (36%), leaving us without information for 4 (5%). Of the 70 journals for which we have information, 53 (76%) will review/accept preprinted manuscripts; 11 (16%) do not, and 6 (9%) are unclear or make decisions on a case-by-case basis. (For a link to our list of HPE journals and our understanding of their policies regarding preprinted manuscripts, see https://jahse.med.utah.edu/submission/ and select “Where to Publish”.) No wonder there is confusion.

We encourage our colleagues across the health professions to join our call to eliminate this confusion by encouraging all HPE journals to support and promote preprinting. The value of preprinting has only become more important during the COVID-19 pandemic [3]. Being able to preprint scholarship prior to formal submission enhances formative review and revision, augments the benefits of peer coaching, and promotes higher quality publications. Preprinting also makes work available to others more quickly, which can enhance collaboration and uptake of new ideas without compromising the eventual copyright of the final published product.
